# Can the Executive Control Network be Used to Diagnose Parkinson's Disease and as an Efficacy Indicator of Deep Brain Stimulation?

**DOI:** 10.1155/2020/6348102

**Published:** 2020-02-14

**Authors:** Wenwen Dong, Chang Qiu, Xu Jiang, Bo Shen, Li Zhang, Weiguo Liu, Wenbin Zhang, Jiu Chen

**Affiliations:** ^1^Department of Functional Neurosurgery, The Affiliated Brain Hospital of Nanjing Medical University, Nanjing, Jiangsu 210029, China; ^2^Department of Geriatric Medicine, The Affiliated Brain Hospital of Nanjing Medical University, Nanjing, Jiangsu 210029, China; ^3^Neurology Department, The Affiliated Brain Hospital of Nanjing Medical University, Fourth Clinical College of Nanjing Medical University, Nanjing, Jiangsu 210029, China; ^4^Institute of Neuropsychiatry, The Affiliated Brain Hospital of Nanjing Medical University, Fourth Clinical College of Nanjing Medical University, Nanjing, Jiangsu 210029, China; ^5^Institute of Brain Functional Imaging, Nanjing Medical University, Nanjing, Jiangsu 210029, China

## Abstract

**Objective:**

The aim of this work was to investigate whether there are differences in the executive control network (ECN) between patients with Parkinson's disease (PD) before and after deep brain stimulation (DBS) surgery and to explore how deep brain stimulation (DBS) surgery affects ECN connectivity in patients with PD.

**Methods:**

Resting-state magnetic resonance imaging (MRI) data were obtained from 23 patients with Parkinson's disease preoperatively (pre-PD) and postoperatively (post-PD) and 14 normal controls (CN). The right dorsolateral prefrontal cortex (DLPFC) was used as the seed region of interest (ROI) to study the characteristics of the functional connectivity of the ECN in these subjects.

**Results:**

There were differences in the ECN among PD patients before and after surgery and between the CN. Compared with the CN group, the pre-PD patients showed significantly reduced functional connectivity (FC) between the DLPFC and the left inferior frontal gyrus, left precuneus, left cerebellum posterior lobe, right middle frontal gyrus, right inferior parietal gyrus, right posterior central gyrus, right precuneus, and right inferior frontal gyrus. Compared to the CN group, the post-PD patients showed significantly reduced FC between the DLPFC and left inferior frontal gyrus, left precuneus, left cerebellum posterior lobe, right middle frontal gyrus, right inferior frontal gyrus, and right parietal lobule. There is no difference in the ECN between the pre-PD patients and the post-PD patients.

**Conclusions:**

The FC of ECN in PD patients was different from that in normal controls, but the FC of the ECN in patients with PD may not be altered by DBS. This suggests that the ECN may be considered an imaging biomarker for the identification of PD but may not be a good imaging biomarker for the evaluation of DBS efficacy.

## 1. Introduction

Parkinson's disease (PD) is a common neurodegenerative disease in middle and older age adults, second in incidence to Alzheimer's disease [1]. The incidence of the disease increases with age, typically developing between the age of 55 and 65 [2, 3]. PD presents with both motor symptoms and nonmotor symptoms, of which motor symptoms mainly manifest as quiescent tremors, slow movements, myotonia, and postural balance disorders, while nonmotor symptoms mainly manifest as cognitive impairment (such as verbal, attention, and executive function), emotional disorders, sleep disorders, and autonomic nervous dysfunction. Among them, the effect of executive function (EF) disorder on the activities of daily living of PD patients has received increased attention [4, 5]. EF is defined as the cognitive process involved in goal-directed behavior from goal formulation and intention formation to successful execution and processing of the outcome [6–8]. EF impairment has been found in newly diagnosed PD patients and negatively impacts their ability to participate in the activities of daily living and their quality of life [9–11]. Patients with PD-related executive dysfunction may have difficulty in controlling and regulating their behavior or performing tasks that require attention and complex thinking. Therefore, studying the EF of PD patients and understanding the mechanism of executive dysfunction may provide new information for the diagnosis and treatment of PD.

The treatment of PD, including drug, surgical, and dietary treatments, as well as rehabilitation exercises, is constantly improving. Drug therapy is the first treatment choice for PD patients. However, as the duration of medication increases, the dosage administered is increased over time and a series of symptoms, including wearing-off motor fluctuation, on-off phenomenon, and levodopa-induced dyskinesia can occur, which seriously affect the lives of PD patients [12]. Presently, deep brain stimulation (DBS) is a treatment modality for medically refractory PD. The advantage of DBS is that it does not damage the nerve nuclei, avoiding serious complications, such as cerebral hemorrhage, unilateral limb mild hemiplegia, visual field defects, and sensory disorders, caused by that damage. Furthermore, postoperative symptoms can be controlled by adjusting the stimulation parameters. Jahanshahi and his team found that DBS of patients' subthalamic nuclei (STN-DBS) effectively improved not only the motor function but also the EF of PD [13].

With the development of imaging, resting-state functional magnetic resonance (rs-fMRI) has been widely used to study the structure and function of the brain. This type of MRI requires subjects to close their eyes, relax, try not to think about anything, and not fall asleep. Biswal found that rs-fMRI could obtain the functional network of brain spontaneous activity in the resting state, which has a high degree of overlap with the network related to the task state [14], indicating that the brain activity in the resting state is not meaningless. rs-fMRI can be used to study changes in brain network connectivity in subjects. Among the various forms of the brain network, the executive control network plays an important role in the integration of sensory and memory information, the regulation of cognition and behavior, and also participates in the process of working memory [15, 16]. The ECN is often used to study the functional mechanism of EF changes in patients [17, 18], and several research teams have found a close correlation between EF changes and the executive control network [18–22]. To study whether the ECN of PD patients is different from that of normal people and whether DBS changes the functional connectivity (FC) of PD patients' ECN, we selected the seed-based FC on resting-state fMRI to investigate differences in functional connectivity of the brain networks between pre-PD, post-PD, and CN patients and further analyze how DBS improved the symptoms of PD patients from the perspective of brain functional network connectivity.

## 2. Materials and Methods

### 2.1. Participants

This study was approved by the Human Participants Ethics Committee of the Affiliated Brain Hospital of Nanjing Medical University. Written informed consent was obtained from all participants.

This study initially recruited 50 elderly patients, including 36 PD patients and 14 healthy controls (CN), and obtained their rs-fMRI data, in which each PD patient received a magnetic resonance scan before surgery (no medication) and after 3 months of STN-DBS (no medication and stimulation-off). Among these individuals, 13 PD patients were excluded due to excessive motion artifacts (i.e., cumulative translation or rotation over 2.5 mm or 2.5° and point-to-point average translation or rotation over 0.15 mm or 0.1°) and missing or incomplete MRI data from the pre-PD or post-PD. The remaining 23 PD patients and 14 CN cases were included in the subsequent analysis (Table 1). The inclusion criteria for the patient group were [23, 24] (1) a definite diagnosis of idiopathic Parkinson's disease, (2) no dementia or major mental illness, (3) had parkinsonian motor symptoms or dyskinesias that limited their ability to perform the activities of daily living, (4) despite the receipt of optimal medical therapy, a full dose of drugs, at least compound levodopa and dopamine receptor agonists were used, (5) no surgical contraindications, (i.e., able to remain calm and cooperative during surgery), and (6) willingness and ability to make subsequent visits.

### 2.2. Neuropsychological Assessments

All patients received standardized clinical interviews and comprehensive neuropsychological evaluations by neuropsychologists. The Mini-Mental State Exam (MMSE) and Montreal Cognitive Assessment (MoCA) were used to assess the cognitive and executive function [25]. Hamilton Anxiety (HAMA) and Hamilton Depression (HAMD) scales were used to assess the psychological state of the subjects. Because the participants in the CN group were non-Parkinson's disease patients, the Parkinson's disease-related scales did not apply to them and the Unified Parkinson's Disease Rating Scale Part III (UPDRS-III) and the 39-item Parkinson's Disease Questionnaire (PDQ-39) were used only in the PD group.

### 2.3. MRI Data Acquisition

MRI images were acquired in a 1.5 Tesla GE Medical Systems scanner in the Department of Radiology, Nanjing Brain Hospital. All subjects were instructed to keep their eyes closed, relax their minds, and remain as motionless as possible during the scan. Resting-state functional images (128 volumes) were obtained using a gradient-recalled echo-planar imaging (GRE-EPI) sequence, with a repetition time (TR) = 2000 ms, echo time (TE) = 40 ms, flip angle (FA) = 90°, acquisition matrix = 64 × 64, field of view (FOV) = 240 mm × 240 mm, thickness = 3.0 mm, and voxel size = 3.75 × 3.75 × 3 mm^3^. High-resolution T1-weighted axial images covering the whole brain were acquired by 3D magnetization-prepared rapid gradient-echo (MPRAGE) sequence with the following parameters: TR = 11.864 ms, TE = 4.932 ms, FA = 20°, acquisition matrix = 256 × 256, FOV = 152 mms × 152 mm, thickness = 1.4 mm, number of slices = 112, and voxel size = 0.59 × 0.59 × 1.4 mm^3^.

### 2.4. Image Preprocessing

The preprocessing steps have been described in previous studies [26]. All data analyses were processed using SPM8 software (available at http://www.fil.ion.ucl.ac.uk/spm). The first five volumes of the scanning session were discarded to allow for T1 equilibration effects. Corrections were performed for the intravolume acquisition time differences among slices and intervolume motion effects during the scan. The fMRI data were spatially normalized to a standard EPI template and were resampled to 3 × 3 × 3 mm^3^ voxels. Several nuisance variables, including the linear trend and constant, six head motion parameters, the cerebrospinal fluid signal, and the white matter signal, were removed by multiple linear regression analyses. Temporal bandpass filtering (0.01–0.1 Hz) was applied to reduce the effect of low-frequency drifts and high-frequency physiological noise. Finally, functional images were spatially smoothed with a Gaussian kernel of 6 mm (full width at half maximum, FWHM). As described in previous studies [27, 28], to reduce the effect of head movement on the rs-fMRI results, we adopted strict quality assurance measures to ensure that there was no significant difference in head motion quality parameters between the groups.

### 2.5. Functional Connectivity Analyses

The seed region of interest (ROI), indicated by drawing 6 mm spheres located in the right dorsolateral prefrontal cortex (DLPFC, MNI space: 48, 12, and 34) [26], was determined by converging evidence from previous studies. The DLPFC has been consistently considered as a key region within the ECN [26, 29–31]. The individual averaged time series for all voxels within the DLPFC region were extracted as the reference time course. Then, a voxel-wise cross-correlation analysis was conducted between the averaged time courses of all voxels within the seed region and whole brain within the gray matter (GM) mask [26]. A Fisher's *z*-transformation was then applied to improve the normality of the correlation coefficients. For each subject, we obtained one *z*-score map that represented the intrinsic FC patterns of the ECN.

## 3. Statistical Analyses

### 3.1. Demographic and Neuropsychological Data

Two-sample *t*-tests and chi-squared tests (applied only in the comparisons according to gender) were used to test the differences in the demographic data between the PD patients and the CN cases.

### 3.2. Group-Level ECN Intrinsic Connectivity Analysis

The individual spatial maps of FC in each group (CN, pre-PD, and post-PD) were submitted to a random-effect analysis using a one-sample *t*-test with a stringency threshold of *p* < 0.001 using false discovery rate (FDR) correction, together with a cluster extent *k* > 30 voxels (810 mm^3^) to highlight the patterns of ECN intrinsic connectivity in the PD patients and CN subjects.

Within the ECN mask, group differences between pre-PD and CN groups and between post-PD and CN groups were examined using the independent two-sample *t*-test with age and gender treated as covariates, and group differences between pre-PD and post-PD were examined using a paired *t*-test in IBM SPSS version 23. All results were corrected by FDR with a stringency threshold of *p* < 0.01 and a cluster *k* > 30 voxels (810 mm^3^).

## 4. Results

### 4.1. Demographic and Neuropsychological Characteristics

There was no statistically significant difference between the PD group and the CN group in age and gender (*p* > 0.05, Table 1). Compared to the CN group, there were statistically significant differences in MoCA, HAMA, and HAMD scale scores of the PD patients, indicating a certain degree of cognitive decline in PD patients, as well as manifestations of anxiety and depression.

### 4.2. Functional Connectivity Patterns

**Figure 1:**
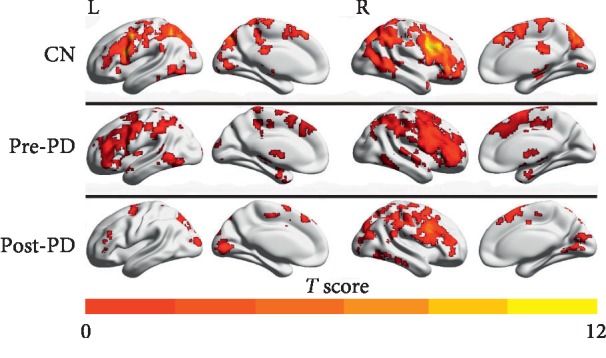
Functional connectivity of the brain network patterns for CN, pre-PD, and post-PD using resting-state functional magnetic resonance imaging (rs-fMRI). Group-level analyses led to three intrinsic connectivity maps. The results are presented using FDR corrected *p* < 0.001 and cluster extent *k* > 30 voxels (810 mm^3^). Abbreviations. CN, normal controls; pre-PD, preoperation Parkinson's disease; post-PD, postoperation Parkinson's disease; ECN, executive control network; L, left; R, right.

Figure 1 shows the intrinsic connectivity mapping between the groups using FDR correction *p* < 0.001 and clustering range *k* > 30 voxels (810 mm^3^).

### 4.3. Comparison of Connectivity Patterns among Pre-PD, Post-PD, and CN

Table 2 shows the brain regions of difference between the preoperative and postoperative executive control networks and the normal control group.

**Figure 2:**
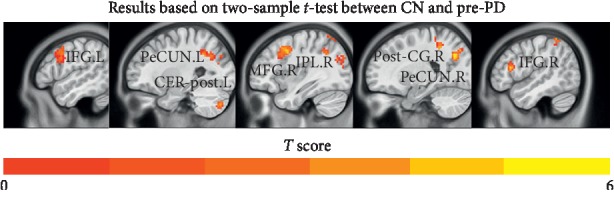
Comparisons of functional connectivity between CN and pre-PD (*p* < 0.01, FDR corrected and cluster extent *k* > 30 voxels). Abbreviations: CN, normal controls; pre-PD, preoperation Parkinson's disease; ECN, executive control network; IFG.L, left inferior frontal gyrus; PeCUN.L, left precuneus; CER-post.L, left cerebellum posterior lobe; MFG.R, right middle frontal gyrus; IPL.R, right inferior parietal lobule; Post-CG.R, right postcentral gyrus; PeCUN.R, right precuneus; IFG.R, right inferior frontal gyrus.

As shown in Figure 2 and Table 2, compared to the CN group, the pre-PD group showed significantly reduced FC in the ECN between the DLPFC and the left inferior frontal gyrus, left precuneus, left cerebellum posterior lobe, right middle frontal gyrus, right inferior parietal gyrus, right posterior central gyrus, right precuneus, and right inferior frontal gyrus.

**Figure 3:**
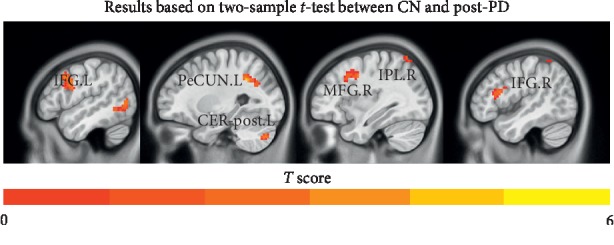
Comparisons of functional connectivity between CN and post-PD (*p* < 0.001, FDR corrected and cluster extent *k* > 30 voxels). Abbreviations: CN, normal controls; post-PD, postoperation Parkinson's disease; ECN, executive control network; IFG.L, left inferior frontal gyrus; PeCUN.L, left precuneus; CER-post.L, left cerebellum posterior lobe; MFG.R, right middle frontal gyrus; IPL.R, right inferior parietal lobule; IFG.R, right inferior frontal gyrus.

As shown in Figure 3 and Table 2, compared to the CN group, the post-PD group showed significantly reduced FC in the ECN between the DLPFC and left inferior frontal gyrus, left precuneus, left cerebellum posterior lobe, right middle frontal gyrus, right inferior frontal gyrus, and right parietal lobule.

Interestingly, as shown in Table 2, there is no difference in the ECN between the pre-PD patients and the post-PD patients.

## 5. Discussion

In the early course of the disease, PD patients begin to show signs of impaired executive function and their ability and quality of life are significantly lower than those of people without PD. Their movements gradually slow down, and they appear to be inattentive. In addition, their information processing speed slows down and their spatial episodic memory declines. These clinical manifestations are consistent with our findings (Table 1 and Figure 2). In the executive network connection of PD patients, the DLPFC has weakened functional connections with parts of the brain areas, such as the frontal cortex, precuneus, and posterior central gyrus, which are related to episodic memory, self-related information processing, consciousness, executive ability, and emotional evaluation. Presently, a PD diagnosis is mainly based on the patient's motor symptoms and the doctor's experience and the early diagnostic accuracy is very low [32, 33]. Based on the results of this study, we speculate that the executive control network may provide additional information for the diagnosis of PD and that the executive control network has a potential research value as an imaging biomarker of PD.

Deep brain stimulation is clearly one of the most effective ways to provide relief to patients suffering from Parkinson's disease. With continuous stimulation of the intracranial electrodes, the patients' symptoms improve and they are able to walk normally and participate in other normal activities. However, the significant improvement of postoperative patients' symptoms cannot be explained by the results given in Table 2. That is, there is no difference in the ECN between the pre-PD patients and the post-PD patients. In addition, by comparing postoperative PD patients with the normal control group, the connection strength between the DLPFC and the right inferior frontal gyrus, right middle frontal gyrus, right parietal lobe, left inferior frontal gyrus, and left anterior wedge lobe was still reduced. We hypothesized that the need to briefly turn off the patient's electrode stimulation during the collection of postoperative MRI data may have skewed the results, or the FC of the ECN in patients with PD may not be altered by DBS. One of the limitations of this study was that the electrodes needed to be turned off to avoid damaging the patient with a strong magnetic field. Another limitation was that the lack of an EF test for postoperative patients to validate the patient's ECN results.

In summary, this study demonstrated that the ECN of the resting state in PD patients was different from that of the normal control group and the decrease in functional connections in related brain areas was consistent with the clinical manifestations of PD. ECN plays an important role in the integration of sensory working memory information and the regulation of behavior. This suggests that the ECN may be considered as a potential imaging biomarker for the diagnosis of PD. As for the effect of DBS, when PD patients are undergoing resting functional magnetic resonance, the executive control network does not exhibit changes in the brain areas related to the executive control. Due to the aforementioned limitations, ECN may not be a good imaging marker for evaluating the efficacy of deep brain stimulation. Of course, we may be able to further explore the potential mechanisms of PD disease and DBS stimulation to improve PD symptoms by combining other network task models (e.g., fMRI-based small-world network, default mode network, and other model data) and other means (e.g., EEGs and magnetoencephalograms).

## Figures and Tables

**Table 1:** Demographics and clinical measures of patients with PD and controls.

Items	CN	PD	T values (*χ*^2^)	*p* values
	*n* = 14	*n* = 23		
Age (years)	63.29 (9.72)	60.91 (12.62)	1.134	0.323
Gender (male/female)	7/7	9/14	0.419	0.517
MMSE	27.86 (1.86)	26.57 (2.37)	1.733	0.092
MoCA^*∗*^	28.71 (1.07)	24.91 (2.39)	5.593	<0.001
HAMA^*∗*^	0.43 (0.51)	5.00 (3.81)	4.436	<0.001
HAMD^*∗*^	0.79 (0.89)	5.26 (4.17)	3.961	<0.001
UPDRS-III	—	39.30 (12.47)	—	—
PDQ-39	—	44.09 (17.29)	—	—

Data are presented as the mean (with standard deviation, SD). Abbreviations: CN, controls; PD, Parkinson's disease; MMSE, Mini-Mental State Exam; MoCA, Montreal Cognitive Assessment scale; HAMA, Hamilton Anxiety Scale; HAMD, Hamilton Depression Scale; UPDRS-III, Unified Parkinson's Disease Rating Scale Part III; PDQ-39, the 39-item Parkinson's Disease Questionnaire. ^*∗*^Significant differences were found between the two groups. The *p* values were obtained by using the independent sample *t* test except for gender (chi-square test).

**Table 2:** Comparisons of functional connectivity of the brain networks during CN, pre-PD, and post-PD.

Brain regions	Peak MNI coordinate			Peak *T* value	Cluster size (mm^3^)
	*x*	*y*	*z*		
*CN* *>* *pre-PD*					
R inferior frontal gyrus	48	24	15	5.44	1269
L precuneus	−27	−51	36	4.79	4698
R precuneus	21	−66	36	6.18	5967
L inferior frontal gyrus	−57	6	39	5.29	2430
R middle frontal gyrus	36	9	42	5.26	2862
R postcentral gyrus	24	−45	51	5.58	1350
R inferior parietal lobule	36	−57	36	4.72	2727
L cerebellum posterior lobe	−30	−78	−45	5.09	1458

*CN* *>* *post-PD*					
L cerebellum posterior lobe	−30	−78	−42	5.27	1107
R inferior frontal gyrus	45	18	18	4.11	918
L inferior frontal gyrus	−57	6	39	5.60	4509
L precuneus	−27	−51	36	5.13	1809
R middle frontal gyrus	33	3	39	4.08	1377
R inferior parietal lobule	39	−69	54	4.33	1080

*Pre-PD* *<* *post-PD*					
Nr	—	—	—	—	—

Abbreviations: CN, controls; pre-PD, preoperation Parkinson's disease; post-PD, postoperation Parkinson's disease; ECN, executive control network; Nr, no statistically significant differences between brain regions.

## Data Availability

The authors declare that underlying data of this research are available within the article.

## Supplementary Materials

Supplementary Materials The final supplementary materials file records the clinical measures of patients with PD and controls, including the participants' gender, age, and clinical scale scores. .
